# Investigation of MicroRNAs as predictors of radioligand therapy response in gastroenteropancreatic neuroendocrine tumours

**DOI:** 10.1038/s41598-026-40046-z

**Published:** 2026-02-17

**Authors:** Federica Scalorbi, Enrico Matteo Garanzini, Chiara Marzi, Manuela Gariboldi, Giuseppina Calareso, Michela Baccini, Giovanna Sabella, Sara Pusceddu, Alfonso Marchianò, Luca Roz, Massimo Milione, Marco Maccauro

**Affiliations:** 1 Nuclear Medicine Department, ENETS Center of Excellence, Fondazione IRCCS Istituto Nazionale dei Tumori, Milan, Italy; 2 Department of Radiodiagnostics and Radiotherapy, ENETS Center of Excellence, Fondazione IRCCS Istituto Nazionale Tumori, Milan, Italy; 3 Department of Statistics, Computer Science, Applications “G. Parenti”, University of Florence, Viale Morgagni 59, Florence, 50134 Italy; 4 Molecular Epigenomics Unit, Department of Experimental Oncology, ENETS Center of Excellence, Fondazione IRCCS Istituto Nazionale dei Tumori, Milan, Italy; 5 First Division of Pathology, Department of Pathology and Laboratory Medicine, ENETS Center of Excellence, Fondazione IRCCS Istituto Nazionale dei Tumori, Milan, Italy; 6 Department of Medical Oncology, ENETS Center of Excellence, Fondazione IRCCS Istituto Nazionale dei Tumori, Milan, Italy; 7 Tumor Genomics Unit, Department of Experimental Oncology, ENETS Center of Excellence, Fondazione IRCCS Istituto Nazionale dei Tumori, Milan, Italy

**Keywords:** Biomarkers, GEP-NETs, MiRNA, qRT-PCR, Response to treatment, RLT, Biomarkers, Cancer, Oncology

## Abstract

Gastroenteropancreatic neuroendocrine tumours (GEP-NETs) are commonly treated with radio-ligand therapy (RLT) but reliable biomarkers for predicting early disease progression are lacking. In this exploratory study we investigated whether microRNA (miRNA) expression in archival samples is associated with early response to RLT and with tumour origin and grading. Forty-eight formalin-fixed, paraffin-embedded samples from G1–G2 GEP-NETs patients treated with RLT (^177^Lu-Oxodotreotide (Lutathera^®^) were analyzed. Two CT or MRI scans per patient, performed within three months before and after RLT, were used to assess early progression (PD) according to RECIST v1.1. Thirteen miRNAs previously implicated in NET biology were quantified by qRT-PCR. Multiple logistic regression evaluated associations between miRNA expression and early progression, as well as relationships with tumour origin and grade. We observed trends suggesting that lower expression of miR-21-5p (OR = 0.51, 90% CI: 0.26–1.00) and miR-196a (OR = 0.78, 90% CI: 0.59–1.02), and higher expression of miR‑30a-5p (OR = 2.62, 90% CI: 0.1.14–6.05) may be associated with a reduced likelihood of early progression. Lower miR-196a and higher miR‑30a-5p expression was also more frequent in pancreatic tumours and lower miR-196a expression is associated to G1 lesions. These findings indicate that miRNA profiling in GEP-NETs archival samples is feasible and support the potential role of miR-21-5p, miR-196a, and miR‑30a-5p as exploratory biomarkers for early progression afterRLT. Further validation in independent cohorts is required to confirm these preliminary observations.

**Supplementary Information:**

The online version contains supplementary material available at 10.1038/s41598-026-40046-z.

## Introduction

MicroRNAs (miRNAs or miRs) are small non-coding RNAs of 19–24 nucleotides, first discovered in Caenorhabditis elegans^1^. They regulate gene expression post-transcriptionally by inhibiting the translation of target mRNAs, acting as either tumour suppressors or oncogenes depending on the gene context^2^. A single miRNA can target multiple mRNAs and influences processes such as apoptosis, proliferation, invasion, drug resistance and epithelial-to-mesenchymal transition^3–5^. MiRNAs are stable molecules, allowing robust quantification in fresh-frozen and FFPE tissues, as well as in biological fluids^6^. Dysregulated miRNA expression has been implicated in neuroendocrine tumours (NETs), including gastroenteropancreatic NETs (GEP-NETs)^7–10^.

Neuroendocrine neoplasms (NENs) are a heterogeneous group of tumours which arise from neuroendocrine cells andcan originate in multiple organs. Among them, GEP-NETs are rare neoplasms that arise within the gastrointestinal tract. Their reported incidence has increased over recent decades, mainly due to improvements in early-stage diagnosis^11^. Currently, GEP-NETs represent the second most prevalent type of gastrointestinal tumour after colorectal cancer^12^. Well-differentiated G1-G2 GEP-NETs exhibit relatively indolent behaviour when localized and resectable. There are many possible therapeutic protocols (localized or systemic), in accordance with disease burden, but two are the cornerstones of GEP-NETs therapy: primary tumour surgical removal and somatostatin analogue (SSA) therapy^13–15^. However, predicting progression, therapeutic response and survival remains challenging. Ki67/MIB1 proliferative index provides prognostic information but suffers from inter-reader variability and intra- and inter-lesion heterogeneity, which affect treatment outcomes^13,16,17^. Although radio-ligand therapy (RLT) dosimetry may predict response^18^, it is not widely applied due to technical constraints.

Considering the aforementioned, recent studies have explored biomarkers for NETs, including Chromogranin A (CgA), NETest, or combinations of imaging and circulating transcripts^19,20^. Although CgA is the most widely used circulating biomarker, its clinical utility is limited by suboptimal sensitivity and specificity and confounding factors such as proton pump inhibitor use or renal dysfunction^20^.

These limitations have prompted investigation of additional markers, including circulating and tissue miRNAs. MiRNA profiling has been used to aid differential diagnosis, complementary to WHO grading, and to evaluate metastatic spread^21–25^. Few studies have assessed miRNAs as predictors of response to systemic therapies, such as SSAs, or patient survival^21,22,25–29^. Recently, Kövesdi et al. showed that miRNAs can complement CgA, improving diagnostic accuracy when CgA alone is inconclusive^30^. A review by Geisler et al. highlighted that multiple miRNAs, rather than a single marker, are needed to capture the complex biology of NETs, reflecting their roles in proliferation, apoptosis, and metastatic potential^31^.

To our knowledge, no studies have evaluated the association between miRNA expression in FFPE samples and response to RLT. Identifying predictive biomarkers could improve patient selection and enable a more personalized therapeutic approach. We therefore hypothesized that specific miRNA levels sould predict response to RLT, distinguishing patients with early disease progression (PD) from those with more favourable outcomes (non-PD).

## Results

Patient population characteristics are illustrated in Table 1. A total of 48 FFPE samples from 28 patients were retrospectively analyzed. The cohort was predominantly female (67.9%), with a midgut GEP-NET (66.7%), and graded as G2 (60.4%) in accordance with WHO 2019. In most of cases the sample was obtained from ileum and liver (29.2% in both cases), followed by the lymph nodes (22.9%) and pancreas (14.6%). Eleven (nearly 40%) patients exhibited early progression on radiological evaluation after RLT, performed, on average, 18.47 months after the end of treatment (range: 1.38–40.14 months). Four out of 28 patients did not complete RLT due to disease progression. One out of these four patients received 2 RLT administration (7.4 GBq each), the remaining three administrations.

**Table 1:** Description of the study population (*n* = 28 patients for a total of 48 samples).

Category	Data	Value
Patient cohort	Total number	28
	Females (%)	19 (67.9)
	Males (%)	9 (32.1)
Age (years)	Mean (SD)	63.46 (11.40)
RLT Response	PR (%)	1 (3.6)
	SD (%)	15 (53.6)
	PD (%)	11 (39.3)
	Missing	1 (3.6)
Time to follow-up (months)*	Mean (SD)	17.12 (13.37)
Sample cohort	Total number	48
Primary Tumour**	Midgut (%)	32 (66.7)
	Foregut (%)	16 (33.3)
Sample site	Pancreas (%)	7 (14.6)
	Limph node (%)	11 (22.9)
	Liver	14 (29.2)
	Ileum	14 (29.2
	Other metastasis	2 (4.2)
Metastatic sample	Total Number (%)	25 (52.1)
WHO Grading	G1 (%)	19 (39.6)
	G2 (%)	29 (60.4)

*Information is missing for 3.6% of the patients. PD: progressive disease; PR: partial response; RLT: radioligand therapy; SD: stable disease.

**Samples of pancreatic origin have been considered as belonging to the foregut group, in accordance with the embryological origin of the pancreas.

Following an extensive literature review, 13 miRNAs were selected based on their expression in neuroendocrine tumours, where expression varied according to WHO grading, anatomical site of the primary tumour, presence of metastatic lesions or patient prognosis/survival. The list of the selected miRNAs, together with their literature source, are summarized in Table 2. The 13 miRNAs were tested by quantitative real-time PCR (qRT-PCR) on a subset of 16 FFPE samples, revealing that 4 miRNAs (miR‑1264, miR‑488, miR‑1284, miR‑491) exhibited very low expression. Consequently, only the remaining 9 microRNAs were included in the analysis of the full cohort, across all the 48 samples. The normalized expression of the 9 microRNA, calculated by the ΔΔCt formula, has been reported in Supplementary Table S1.

**Table 2:** Overview of the correlation between the expression levels of nine MicroRNAs selected from the literature and their associated biological functions in neuroendocrine tumours.

MicroRNA	Biological/Clinical Function
*miR-133a*	Downregulated in samples from primary tumour to distant metastasis^71^.
*miR-96*	Increased expression during progression from primary to metastatic GI-NENs^71^.
*miR-30a-5p*	Overexpressed in distant metastasis; strong correlation with proliferation index^25^.
*miR-21-5p*	Overexpressed in many tumours, specifically in metastatic high Ki67 pancreatic NET. Programmed cell death 4 (PDCD4) gene is a tumour suppressor involved in cellular invasion and is putatively targeted by miR-21. PDCD4 expression is lost in progressed carcinomas^35^.
*miR-101*	Tumour suppressor that inhibits colony formation, migration, invasion, and lymph node metastasis^72^, cell proliferation^73^, migration and invasion targeting the small GTPase Rac1. Its expression is downregulated in cancers^45^.
*miR-34*	A suppressive microRNA that has a synergistic effect with p53; it is dysregulated in various human cancers^74^.
*miR-210*	Induced by hypoxia-inducible factor 1-alpha (HIF1α); plays a role in adaptation to hypoxia-induced stress. Overexpressed during liver metastasis progression, particularly in pancreatic NETs^75^.
*miR-375*	Downregulated in liver metastases and associated with shorter overall survival^21^.
*miR-196a*	Correlated with advanced stage, lymph node metastases, higher mitotic count, and elevated Ki67 index. Overexpressed in poor prognosis cases (overall survival and disease-free survival)^36^.

**Fig. 1:**
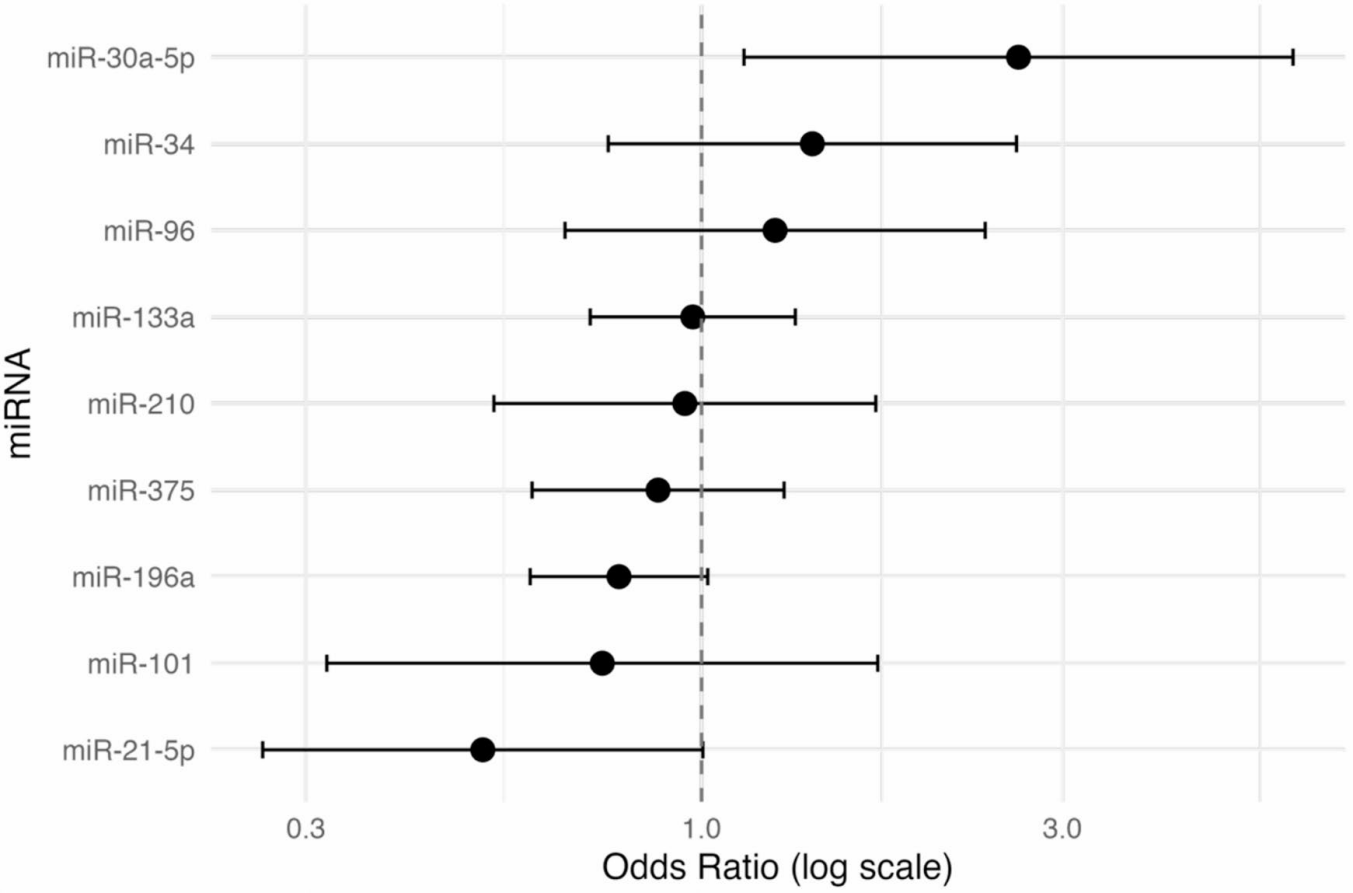
Forest plot of the OR (in the log scale for symmetry around the null value) along with the 90% confidence interval of the logistic model for early PD prediction.

To assess whether the expression levels of nine candidate miRNAs were associated with response to RLT, we fitted a logistic regression model on a dataset obtained through multiple imputation. Three miRNAs, miR-30a-5p, miR-21-5p, and miR-196a, showed a trend towards association with therapeutic response. Specifically, higher values of ΔΔCt for miR-30a-5p were associated with higher odds of non-response (odds ratio (OR) = 2.624, 90% CI: 1.1379–6.0509) (Table 3). According to the ΔΔCt method, higher ΔΔCt values correspond to lower microRNA expression. Thus, the under expression of miR-30a-5p seems associated with a higher probability of non-response to RLT. Contrary, higher values of ΔΔCt for miR-21-5p and miR-196a were associated with lower odds of non-response (OR = 0.5139, 90% CI: 0.2630–1.0041; OR = 0.7778, 90% CI: 0.5936–1.0190 respectively) (Table 3; Fig. 1). In other words, since both miR-21-5p and miR-196a are oncogenes, their overexpression promotes tumour progression and, consequently, can be predictive of poor response to treatment and vice versa^32,33^. The results of the sensitivity analyses are presented in Supplementary Table S2. Overall, the findings related to progressive disease were consistent with those of the primary analysis, again identifying miR-21-5p, miR-196a, and miR-30a-5p as potential biomarkers of treatment response.

**Table 3:** Logistic regression for RLT response prediction. An increase in ΔΔCt corresponds to a decrease in MiRNA expression. Non-PD is the reference group.

MicroRNA	OR	90% CI
*miR‑21-5p*	0.5139	0.2630–1.0041
*miR‑375*	0.8761	0.5971–1.2856
*miR‑196a*	0.7778	0.5936–1.0190
*miR‑30a-5p*	2.624	1.1379–6.0509
*miR‑96*	1.2509	0.6600–2.3708
*miR‑101*	0.7393	0.3197–1.7096
*miR‑34*	1.4011	0.7528–2.6075
*miR‑133a*	0.9737	0.7127–1.3302
*miR‑210*	0.9505	0.5316–1.6996

CI: confidence interval; OR: odds ratio.

These three miRNAs were subsequently included in a logistic regression model to evaluate their association with tumour origin (midgut vs. foregut), considering only samples derived from metastatic tissue. There is evidence that foregut tumours have a tendency toward smaller expression levels of miR-196a (OR = 2.2463, 90% CI: 1.1716–4.3069) and higher expression levels for miR-30a-5p (OR = 0.0732, 90% CI: 0.0064–0.8394), compared to midgut tumours (Table 4).

**Table 4:** Logistic regression for origin (using only the 25 metastatic samples, midgut as reference) and grading (G1 as reference) prediction. An increase in ΔΔCt corresponds to a decrease in MiRNA expression.

MicroRNA	OR	90% CI
Origin (midgut vs. foregut)		
miR21-5p	3.2379	0.7066–14.837
miR196a	2.2463	1.1716–4.3069
miR30a-5p	0.0732	0.0064–0.8394
Grading (G1 vs. G2)		
miR21-5p	1.03	0.7122–1.7092
miR196a	0.5483	0.4016–0.7488
miR30a-5p	0.8677	0.5225–1.4408

CI: confidence interval; OR: odds ratio.

Lastly, the same three miRNAs were tested for association with tumour grade (GI vs. G2). In this model, there is evidence that over expression of miR-196a was associated with G1 tumour grade (OR = 0.5483, 90% CI: 0.4016–0.7488) (Table 4).

## Discussion

The aim of this study was to explore the potential association between miRNA expression in archival FFPE GEP-NETs samples and early response to RLT, with a focus on early disease progression. Our results suggest that three miRNAs, miR-21-5p, miR-196a and miR‑30a-5p, may be associated with treatment outcome, as downregulation of miR-21-5p and miR-196a and overexpression of miR‑30a-5p was associated with an increased likelihood of response to RLT.

Based on the available literature, evidence on potential associations between miRNA expression and response to systemic treatment, as SSA, is limited to a single review^28^. Considering response to RLT, only a study has directly investigated this topic, reporting a potential association between circulating microRNA expression and response to treatment^34^in pancreatic NETs. In details, they investigated on plasma samples a distinct set of microRNAs from those analysed in our study and identified only one circulating miRNA, the hsa-miR-5096. This miRNA was reported as a potential biomarker of outcome in patients undergoing RLT, being associated with both 6-month progression-free survival (PFS) and 12-month OS. Moreover, its expression has been reported to identify ^18^F-FDG-PET/CT–positive pancreatic NETs, which are known to be characterized by a more aggressive biological behaviour and a worse prognosis following RLT.

In our study a specific analysis of metastatic samples showed that lower miR-196a expression was more frequently associated with foregut origin and G1 grading, supporting previous evidence linking miRNA profiles to proliferative index and primary site in GEP-NETs^21,25,35^. Furthermore, a study by Lee et al.^36^, investigating the prediction of recurrence risk in surgically resected pancreatic NETs, showed that miR-196a overexpression was significantly associated with advanced pathological stage of both primary tumour and lymph node metastases, and was indicative of poorer prognosis in terms of overall survival (OS) and disease-free survival (DFS). Additionally, in our study, miR-30a-5p was downregulated in patients who experienced disease progression after RLT, consistent with its proposed tumour-suppressive role in several cancers^37–40^. However, this contrasts with Zimmermann et al., who reported higher miR-30a-5p expression in metastatic samples, compared with non-metastatic ones. Interestingly, in line with Zimmermann et al., we also observed higher miR-30a-5p expression in foregut tumours compared to midgut^25^. In addition, our study identified a differential expression of miR-21-5p, a well-known oncomiR that targets PDCD4 (Programmed Cell Death 4 gene). MiR-21 has been investigated in GEP-NETs by Roldo et al.^35^, who confirmed PDCD4 as a potential target of miR-21 in pancreatic neoplasms. MiR-21 can also control PTEN, a well-known tumour suppressor, promoting tumorigenesis acting on the PI3K –AKT-mTOR tumour suppressor pathway. In line with this evidence, the downregulation of PDCD4 and PTEN has been shown to contribute to tumour cell proliferation in several malignancies^41^. Furthermore, in the context of neuroendocrine tumours, previous studies have reported that miR-21 and miR-600 are overexpressed in primary GEP-NETs with metastatic disease with their expression levels correlating with the Ki-67 proliferation index^25^. In line with these findings, miR-21 upregulation has been associated with proliferative pNETs and poor overall survival^42^and has been shown to regulate FOXM1 via the PI3K pathway, a known key signalling axis involved in tumour progression and poor prognosis^21,43^. Other microRNAs have been reported in literature to correlate with the primary tumour origin. For example, MiR-375, a known tumour suppressor highly expressed in well-differentiated GEPNETs^23^, that showed no association with RLT response in our study. In a similar approach, we evaluated the expression of additional microRNAs (miR-133a, miR-96, miR-101, miR-34 and miR-210), which have been previously reported to be involved in tumour progression, metastasis and hypoxia response^25,40,44–46^. Despite these reported roles, we did not observe any significant associations between their expression and response to RLT in our cohort. Other microRNAs not investigated in this study, as miR-1290, have been proposed to distinguish different pancreatic neoplasms, such as pancreatic ductal adenocarcinoma from pancreatic neuroendocrine tumours^47^, and may also serve as prognostic biomarker in neuroendocrine tumour lesions^48^. Taken together, these results support the potential of miRNA profiling - particularly for miR-196a, miR-30a-5p and miR-21-5p - as a tool for tumour stratification and identification of tissue of origin^23,24^.

This study has some limitations: the cohort was small, potential confounders including prior therapies were not evaluated and RECIST-based RLT response assessment has recognized limitations. Therefore, the present work should be interpreted as proof-of-concept, demonstrating feasibility and generating hypotheses rather than providing validated predictive biomarkers.

In conclusion, miRNA profiling in archival GEP-NET samples is feasible and may provide preliminary insights into RLT response. Downregulation of miR-21-5p and miR-196a and overexpression of miR‑30a-5p correlated with more favourable outcomes, with miR-196a also associated with tumour origin and grade. Furthermore, evidence suggests that foregut tumours tend to exhibit lower expression levels of miR-196a and higher expression of miR-30a-5p, compared with midgut tumours. These results require validation in larger, independent cohorts and currently serve to guide future studies in patient stratification and personalized RLT approaches, particularly in challenging scenarios such as carcinoma of unknown primary.

## Methods

### Data collection

Clinical data were retrospectively gathered from GEP-NETs patients treated with RLT (^177^Lu-Oxodotreotide (Lutathera^®^) at the IRCCS Foundation, National Cancer Institute of Milan, between May 2019 and December 2021.

Inclusion criteria included adults aged 18 or older, with histopathologically confirmed WHO 2019 G1 or G2 tumours, at least one high‑quality FFPE histological specimen per patient from either the primary tumour or metastatic site (lymph node or distant metastases) and provided contrast-enhanced CT/MRI scans during follow-up to evaluate the response to RLT. Exclusion criteria included absence of primary tumour diagnosis, lack of Ki‑67 index assessment, FFPE sample cellularity lower than 60%, protein or solvent contamination in the extracted RNA, missing radiological imaging or no signed informed consent.

All patients received ^177^Lu-Oxodotreotide (Lutathera^®^) and SSA in compliance with EMA and AIFA guidelines [Lutathera | European Medicines Agency (https://www.ema.europa.eu/en/medicines/human/EPAR/lutathera); AGENZIA ITALIANA DEL FARMACO | DETERMINA 11 marzo 2019. https://www.aifa.gov.it/documents/20142/847786/Determina_501-2019_Lutathera.pdf]. Prior to RLT, tumour burden and somatostatin receptor expression were evaluated using contrast-enhanced CT/MRI and ^68^Ga-DOTA-SSA PET/Octreoscan, respectively, in accordance with clinical practice.

Recorded clinical parameters included age, gender and proliferation index (Ki67%). During follow-up, CT and MRI scans were collected after RLT to evaluate therapeutic response according to RECIST v. 1.1 criteria. Response was assessed categorically as complete response (CR), partial response (PR), stable disease (SD) or PD. For the purposes of our analysis, treatment response was dichotomized (progression vs. non-progression, i.e., PD vs. no PD), with patients achieving CR, PR, or SD grouped under the term non-PD. It is important to note that RECIST v. 1.1 have known limitations in evaluating treatment response. Indeed, the RECIST v1.1 criteria fail to account for alterations in tumour perfusion and treatment-related effects associated with locoregional procedures or anti-angiogenic therapies, leading to underestimation or misinterpretation of lesion response due to edema or necrosis^49,50^. To overcome the limitations of RECIST 1.1, alternative radiological response criteria have been proposed, including the Choi, which integrate changes in both tumour size and attenuation on contrast enhanced CT. Notably, several studies comparing RECIST and Choi criteria in NETs have reported comparable or superior performance of the Choi in treatment response assessment^51–54^. On the other hand, radiological evaluation of NET lesions is commonly performed, by ceCT, during the arterial phase because of their marked early contrast enhancement^55^, whereas Choi criteria are applied to the portal venous phase, limiting their routine clinical applicability. Moreover, both RECIST and Choi provide static evaluations at predefined time points and do not capture tumour dynamics over time. These limitations highlight the need for quantitative and dynamic biomarkers to assess prognosis in GEP-NETs, particularly during systemic treatments such as RLT. Alternatively, tumour growth rate (TGR) provides a dynamic measure of tumour response by estimating volumetric changes over time^56^. Indeed, TGR has been shown to correlate with treatment response and clinical outcomes, including in patients with GEP-NETs^57–62^. A comprehensive review of the literature on the use of TGR in the evaluation of therapeutic strategies in NETs has been provided by Modica et al.^63^. However, conventional TGR calculation assumes spherical tumour geometry, representing a relevant limitation given the frequent presence of irregularly shaped lesions^64^. To overcome this limitation, a previous proof-of-concept study proposed an alternative and more accurate approach - named cylindrical TGR (cTGR) - which better accounts for longitudinal changes in lesion morphology and improves growth estimation in GEP-NET patients treated with RLT^65^. In parallel, nuclear medicine imaging has explored PET-derived quantitative biomarkers for response prediction. In particular, Kratochwil et al. demonstrated that baseline SUVmax of liver metastases on ^68^Ga-DOTATOC PET/CT significantly predicted response probability to RLT in metastatic NET patients, with higher uptake values associated with responding lesions^66^.

### Ethical approval

This observational study received approval from the Ethics Committee of the National Cancer Institute of Milan (protocol INT 6/20) and adhered to the Declaration of Helsinki. All participants provided written informed consent.

### Sample collection and RNA extraction

FFPE histological specimens were retrospectively collected from patients treated with ^177^Lu-Oxodotreotide (Lutathera^®^) at Nuclear Medicine Department, Fondazione IRCCS Istituto Nazionale dei Tumouri, Milan, from 2019 to 2021.

For each specimen, primary tumour, Ki‑67 index (%) and cellularity were reevaluated by two double-blinded expert pathologists. In the event of disagreement, a third expert pathologist was consulted to reach consensus. Only cases with midgut or foregut origin, G1 or G2 grade according to WHO 2019 classification (WHO and IARC, 2019) and a minimum cellularity of 60%, were selected. In accordance with the study inclusion criteria, the analyzed samples exhibited an average cellularity of 80% with a range of 60–95% (data not shown). In this study, microRNA expression was quantified exclusively in archival formalin-fixed, FFPE tumour tissues, rather than in circulating samples.

From each selected paraffin sample, total RNA was extracted, using Maxwell^®^ RSC miRNA Tissue Kit (Promega Corporation, Madison, WI, USA, in accordance with manufacturer’s instructions), performing one biological replicate. The A260/280 and the A260/230 ratios were calculated on extracted RNA as indicators of protein and solvent contamination, respectively (referenced ratio: 1.8-2.0 for A260/280, 2.2 for A260/230).

### Quantitative PCR analysis

RNA was reverse-transcribed into cDNA. The resulting cDNA served as a template in a Reverse Transcription quantitative Polymerase Chain Reaction (RT-qPCR) using TaqMan^®^ MicroRNA Assays applying QuantStudio™ Real-Time PCR Software (Thermo Fisher Scientific, Waltham, MA, USA, according to the manufacturer’s instructions).

The expression of the 13 microRNAs and the two candidates reference housekeeping (RNU48 and U6) was initially assessed in a pilot set of 16 samples. Since four microRNAs (miR-1264, miR-488, miR-1284, and miR-491) showed no detectable expression in any pilot specimens, they were excluded from the full analysis. For each of the remaining miRNAs, both reference housekeepings were evaluated, and their expression stability was assessed by comparing Ct distributions and variability across samples. The reference gene showing the most stable expression for a given miRNA was selected for normalization in the full dataset. Based on this evaluation, RNU48 was used for normalization of most microRNAs, whereas U6 was used for miR‑196a, miR‑21‑5p and miR‑375. The expression of microRNAs and housekeeping was quantified performing three technical replicates per sample, corresponding to three separate RT‑qPCR plates per sample. Each 48 well‑plate contained up to 16 samples, with each miRNA expressed in triplicate and the corresponding housekeeping genes in duplicate. Relative microRNA expression was normalized using the Delta Delta Ct (ΔΔCt) formula, following standard practice. The ΔΔCt is a validated applied method for gene expression quantification, comparing the expression of a target gene and a reference gene in each sample and a corresponding control one. The relative expression of the target gene is calculated, in accordance with Livak et al. formula as $\:{2}^{-\varDelta\:\varDelta\:Ct}$^67^.

### Statistical analysis

Continuous variables were summarized using mean and standard deviation (SD), while categorical variables were described using absolute frequencies and percentages. To account for missing data, we performed multiple imputation using chained equations (MICE)^68^, under a missingness-at-random assumption, applying classification and regression trees (CART) as the imputation method and generating 20 imputed datasets. These datasets were then aggregated into a single complete dataset by averaging continuous variables and using the mode for categorical variables. Subsequently, we fitted a logistic regression model to investigate the potential predictive value of nine candidate miRNAs for response to RLT. As detailed in the Results section, three miRNAs showing a potential association with treatment response were subsequently included as covariates in two additional multiple logistic regression models. These secondary models aimed to assess whether the selected miRNAs were associated with tumour origin (i.e., midgut versus foregut) in metastatic samples only, and tumour grading (G1 versus G2), respectively.

As sensitivity analyses, we additionally fitted a penalised logistic regression with L1 regularisation (LASSO) on the imputed dataset and a logistic regression on the original dataset. The penalized logistic regression with L1 penalty enables both shrinkage and variable selection. The regularisation parameter λ, which controls the strength of the penalty, was selected via generalized cross-validation.

We reported the results in term of OR and 90% confidence interval. With this choice, our intention was to shift the focus toward the magnitude, direction, and uncertainty of the estimated effects - rather than on rigid significance testing - consistent with current recommendations promoting estimation over dichotomous decision-making^69,70^.

The statistical analysis has been performed by using R software, version 4.5.1 (2025-06-13) (R Core Team (2025). R: A Language and Environment for Statistical Computing. R Foundation for Statistical Computing, Vienna, Austria. https://www.R-project.org/*).*

## Supplementary Information

Below is the link to the electronic supplementary material.

Supplementary Material 1

## Data Availability

The datasets generated and/or analyzed during the current study are available from the corresponding author on reasonable request.
