# Supplementary material for: Investigation of MicroRNAs as predictors of radioligand therapy response in gastroenteropancreatic neuroendocrine tumours
Source: Sci Rep. 2026 Feb 17;16:9430. doi: 10.1038/s41598-026-40046-z (PMC13002892; doi:10.1038/s41598-026-40046-z)
Supplement: Supplementary file 1 — Supplementary Material 1 [file 41598_2026_40046_MOESM1_ESM.docx]

**Supplementary material of “Investigation of microRNAs as predictors of radioligand therapy response in gastroenteropancreatic neuroendocrine tumors”**

**Supplementary Table S1. Summary of quantitative expression values (ΔΔCt) for selected miRNAs. ΔΔCt values are dimensionless and reflect relative changes in miRNA expression rather than absolute quantities [1]. Values are presented as mean and standard deviation. Reference are RNU48 or U6 (only for miR-196a, miR-21-5p, and miR-375).**

| **microRNA** | **ΔΔCt expression value (-)** | **Missing, n (%)** | **Non missing, n (%)** |
| --- | --- | --- | --- |
| *miR-21-5p* | 1.72 (2.31) | 0 (0) | 48 (100) |
| *miR-375* | -2.21 (1.92) | 0 (0) | 48 (100) |
| *miR-196a* | 4.77 (2.98) | 13 (27.1) | 35 (72.9) |
| *miR-30a-5p* | 1.09 (1.98) | 0 (0) | 48 (100) |
| *miR-96* | 5.12 (1.68) | 10 (20.8) | 38 (79.2) |
| *miR-101* | 4.22 (1.93) | 3 (6.2) | 45 (93.8) |
| *miR-34* | 2.15 (2.27) | 0 (0) | 48 (100) |
| *miR-133a* | 3.82 (2.69) | 0 (0) | 48 (100) |
| *miR-210* | 3.11 (2.15) | 0 (0) | 48 (100) |

**Supplementary Table S2. Sensitivity analyses: (1) penalised logistic regression with L1 regularisation (LASSO) applied to the imputed dataset; and (2) standard logistic regression applied to the original dataset, both evaluating predictors of response to RLT. Note: an increase in ΔΔCt indicates decreased miRNA expression.**

| **microRNA** | **OR** | **90% CI** |
| --- | --- | --- |
| *Penalised logistic regression with L1 regularisatiion (LASSO) on the imputed dataset* | | |
| *miR‑196a* | 0.8522 | -0.4086 - 0.0000 |
| *miR‑30a-5p* | 1.1996 | 0.0000 - 1.0018 |
| *Logistic regression on the original dataset* | | |
| *miR‑21-5p* | 0.0781 | 0.0088 - 0.6959 |
| *miR‑375* | 0.5827 | 0.2582 - 1.3148 |
| *miR‑196a* | 0.5442 | 0.3220 - 0.9194 |
| *miR‑30a-5p* | 3.3302 | 0.4682 - 23.6889 |
| *miR‑96* | 9.2474 | 0.8920 - 95.8737 |
| *miR‑101* | 0.1813 | 0.0131 - 2.5202 |
| *miR‑34* | 4.6348 | 0.6330 - 33.9382 |
| *miR‑133a* | 1.4271 | 0.7774 - 2.5999 |
| *miR‑210* | 0.8961 | 0.3977 - 2.0193 |
| *miR‑21-5p* | 0.0781 | 0.0088 - 0.6959 |
| *miR‑375* | 0.5827 | 0.2582 - 1.3148 |

CI: confidence interval; OR: odds ratio.

*References*

1. Livak, K. J. & Schmittgen, T. D. Analysis of Relative Gene Expression Data Using Real-Time Quantitative PCR and the 2−ΔΔCT Method. *Methods* **25**, 402–408 (2001).
